# Intra-arterial chemotherapy as primary or secondary treatment for infants diagnosed with advanced retinoblastoma before 3 months of age

**DOI:** 10.1186/s12885-019-5844-5

**Published:** 2019-07-15

**Authors:** Qiuying Chen, Bin Zhang, Yuhao Dong, Xiaokai Mo, Lu Zhang, Jiejun Xia, Jing Zhang, Shuixing Zhang

**Affiliations:** 1 Department of Radiology, the First Affiliated Hospital, Jinan University, No.613, Huangpu West Road, Tianhe District, Guangzhou, Guangdong 510627 People’s Republic of China; 2 Graduate College, Jinan University, No.613, Huangpu West Road, Tianhe District, Guangzhou, Guangdong 510627 People’s Republic of China; 3 Department of Radiology, Guangdong General Hospital/Guangdong Academy of Medical Sciences, Guangzhou, Guangdong People’s Republic of China; 4 Department of Interventional Radiology and Vascular Anomalies, Guangzhou Women and Children’s Medical Center, No.9, Jinsui Road, Tianhe District, Guangzhou, Guangdong 510627 People’s Republic of China

**Keywords:** Intra-arterial chemotherapy, Advanced retinoblastoma, Infants, Survival outcomes, Complications

## Abstract

**Background:**

To evaluate the safety and efficacy of intra-arterial chemotherapy (IAC) for the primary or secondary treatment of infants diagnosed with advanced retinoblastoma before 3 months of age.

**Methods:**

This single-center retrospective study included 39 infants (42 eyes) aged ≤3 months who were diagnosed with unilateral or bilateral advanced intraocular retinoblastoma (group D and E eyes) and received IAC as primary or secondary treatment between June 2012 and February 2017. Based on each patient’s therapeutic history and response to chemotherapeutic drugs, melphalan, topotecan, and/or carboplatin were used for IAC. The main outcomes included the technical success rate for IAC, survival rates, and adverse events.

**Results:**

In total, 29 and 13 eyes received IAC as primary and secondary treatments, respectively. Catheterization was successful in 136 of 137 procedures. All eyes in the secondary IAC group had previously received intravenous chemotherapy. The mean number of IAC sessions for each eye was 3 (range, 2–6). The 2-year ocular survival rates were 80.7% (95% confidence interval [CI], 58.9–91.7) in the primary IAC group and 91.7% (95% CI, 53.9–98.8) in the secondary IAC group. During the follow-up period, 1 patient with unilateral disease (group E) developed extraocular disease and died. The 2-year recurrence-free survival rates in the primary and secondary IAC groups were 71.9% (95% CI, 49.4–85.7) and 75.0% (95% CI, 40.8–91.2), respectively. During each catheterization procedure, the main complications included eyelid erythema (2.4%), fundus hemorrhage (11.9%), myelosuppression (7.7%), transient vomiting and hair loss (2.6%), and transient pancytopenia (2.6%). Prolonged complications included phthisis bulbi (19.0%), vision loss (19.0%), poor vision (9.5%), and cataract (2.4%). There was no case of stroke, neurological impairment, secondary malignant tumor, or metastasis.

**Conclusions:**

Our findings suggest that IAC, whether primary or secondary, is effective and fairly safe for the management of advanced retinoblastoma in infants aged < 3 months. However, adverse events related to intra-arterial injection and the visual outcomes cannot be neglected and require further investigation.

## Background

Retinoblastoma is the most common primary intraocular malignancy in children, affecting approximately 1 in 15,000–20,000 live births worldwide each year [1]. The average age at diagnosis of intraocular retinoblastoma is 18 months: 12 months for bilateral disease and 24 months for unilateral disease. However, with improvements in both parent and physician awareness levels, many children are being diagnosed before 3 months of age [2, 3].

Treatment procedures for infants diagnosed with low-grade tumors in the first 3 months of life include a mix of laser therapy, cryotherapy, and/or plaque brachytherapy. In the past, advanced tumors often required external beam radiotherapy (EBRT). Although the ocular salvage rates were reportedly good [4], short- and long-term complications associated with radiation in such young children remain a concern. In addition to permanent facial abnormalities, secondary malignancies were reported to develop, with the highest occurrence rate in children treated with radiation in the first year of life [5]. As a result, clinicians discontinued the use of radiation therapy for primary management and switched to intravenous chemotherapy (IVC) for children requiring treatment in the first year of life. While this approach has been replicated with success worldwide, insufficient efficacy and concerns about short- and long-term toxicities have directed some specialized centers toward intra-arterial chemotherapy (IAC) by selective infusion through the ophthalmic artery.

IAC allows the achievement of a high concentration of the chemotherapeutic drug in the eye, with minimum systemic toxicity [6]. It is safe and effective for advanced intraocular retinoblastoma, improving the globe salvage rate and minimizing systemic toxicities [7–10]. In addition, it can be used as a secondary treatment for some eyes that do not respond to primary treatments [9]. However, there are concerns regarding its effectiveness in very young infants. In particular, the femoral artery in infants aged < 3 months is only slightly larger than the catheters used. Therefore, catheterization may not be successful or can result in complications associated with intra-arterial injection. Moreover, the dosage of chemotherapeutic drugs, particularly melphalan, is lower for infants aged < 3 months than for older patients. Accordingly, it remains unclear whether this low dosage can adequately destroy tumor cells or increase the possibility of tumor recurrence.

In our center, we routinely use IAC for the management of advanced intraocular retinoblastoma refractory to local therapy alone. The vast majority of publications have reported certain exclusion criteria for the use of IAC in very young infants [8, 11]. However, other than a few reports [12–14] of single cases or small case series involving IAC performed in infants aged < 3 months, no studies have performed detailed evaluations of this treatment for advanced retinoblastoma in such young infants. Accordingly, we conducted the present retrospective study to evaluate the safety and efficacy of IAC as primary or secondary treatment for infants diagnosed with advanced retinoblastoma before 3 months of age in our center.

## Methods

### Patients

This retrospective, single-center study included all infants aged < 3 months who were diagnosed with unilateral or bilateral advanced intraocular retinoblastoma (group D and E eyes) and subsequently received IAC as primary or secondary treatment between June 2012 and February 2017. The study adhered to the tenets of the Declaration of Helsinki, and ethics committee approval was obtained. The exclusion criteria were as follows: extraocular invasion, metastatic disease, or other severe comorbidities before initial treatment; laser photocoagulation, thermotherapy, cryotherapy, plaque radiotherapy, or EBRT before IVC or IAC; follow-up period < 6 months; and use of adjuvant EBRT. The diagnoses were based on ophthalmological examinations performed under anesthesia, with Retcam fundus photographs or magnetic resonance images acquired by an ocular oncologist. The decision to administer IAC was determined by a retinoblastoma treatment team.

We classified the affected eyes according to the International Intraocular Retinoblastoma Classification [15]. The eyes were divided into primary and secondary IAC groups depending on the initial treatment. All IAC procedures were performed in Guangzhou Women and Children’s Medical Center, which boasts of extensive experience in IAC in China. Eyes in the secondary IAC group received several IVC cycles elsewhere and were switched to IAC after an approximate interval of 1 month.

### Treatment

The IVC protocol was similar for all included patients. Each patient was treated with combination triple-drug therapy involving carboplatin, etoposide/teniposide, and vincristine. The dosage was determined according to the patient’s age and weight and the tumor status.

All IAC procedures were performed by experienced interventional radiologists with the patients under general anesthesia. The femoral artery was punctured using the Seldinger technique, and 70–75 IU/kg of heparin was injected for anticoagulation. Then, the internal carotid artery was catheterized with a 4-French catheter. The ophthalmic artery was mapped from the internal carotid artery using serial arteriograms. A 1.5-French microcatheter was selectively inserted at the ostium of the ophthalmic artery under fluoroscopic guidance, and superselective injection was performed to confirm the flow from the ostium into the ophthalmic artery. Based on each patient’s therapeutic history and response to chemotherapeutic drugs, melphalan, topotecan, and/or carboplatin were used for IAC. Diluted chemotherapeutic drugs were directly infused through the microcatheter. The middle meningeal artery was used as an alternative in cases where the ophthalmic artery was difficult to catheterize. Following complete infusion, the microcatheter was withdrawn, and the femoral sheath was removed. Hemostasis in the femoral artery was achieved by manual compression for 10 to 15 min. After an average 12-h observation, the patients were discharged on the same or following day. In patients with advanced disease bilaterally, IAC was simultaneously administered for both eyes. In patients with a low-grade tumor in the opposite eye, IAC was not necessary if focal therapy or IVC could control the tumor. Each IAC cycle was performed at a 4-week interval, and the necessity for further sessions was decided based on the patients’ tumor response, previous treatments, and clinical data such as toxicity development. Enucleation was performed in the case of potential tumor spread (e.g., fundus hemorrhage, neovascular glaucoma, suspicious optic nerve, or suspected extraocular disease on imaging), phthisis bulbi, uncontrolled seeds or retinal detachment, and cataract [16].

### Follow-up

Detailed ophthalmic examinations were performed once a month by the ocular oncologist. During follow-up, each eye was assessed for regression of the solid tumor, vitreous seeds, subretinal seeds, and subretinal fluid. Tumor recurrence was documented. If the tumor showed stability at 3 separate visits, the interval between each examination was extended to 6 months. If recurrent tumor or subretinal/vitreous seeding was identified, focal therapy (e.g., laser photocoagulation, plaque radiotherapy, cryotherapy, intravitreal chemotherapy, and pars plana vitrectomy) was used as adjuvant treatment. In the long term, patients were mainly monitored for the development of second primary tumors.

### Statistical analysis

Survival outcomes included the ocular salvage, overall survival, and recurrence-free survival rates estimated using the Kaplan-Meier method. Basic characteristics included patient factors (sex, age at diagnosis, and first signs), tumor factors (eye stage and laterality), and treatment factors (treatment group, additional treatments, and number of IAC cycles). Ocular and systemic complications were also recorded. All statistical analyses were performed using Stata 12.0 for Windows (Stata, Inc., Chicago, IL, USA). All tests were two-sided, and a *P*-value < 0.05 was considered statistically significant.

## Results

### Patient characteristics

From June 2012 to February 2017, a total of 39 infants (42 eyes) aged < 3 months were diagnosed and treated for advanced intraocular retinoblastoma. IAC was performed as primary and secondary treatment for 29 and 13 eyes, respectively, and unilateral and bilateral disease was observed in 27 and 12 patients, respectively. Among the 12 patients with bilateral disease, 3 exhibited advanced malignancy in both eyes, 2 underwent enucleation of the fellow eye, and 7 exhibited a low-grade tumor in the fellow eye. The median age at diagnosis was 2.0 months (range, 0.3–3.0 months). Leukocoria was the most common initial symptom, followed by strabismus and eyelid erythema. The characteristics of the included patients and eyes are outlined in Table 1.

**Table 1:** Demographic and clinical characteristics of 39 infants with advanced retinoblastoma

	Primary IAC	Secondary IAC
No. of patients	26	13
Age at diagnosis, months		
Median (minimum-maximum)	2.0 (0.3–3.0)	3.0 (0.5–3.0)
Follow-up, months		
Median (minimum-maximum)	20 (6–58)	30 (6–49)
Gender		
Male	15	6
Female	11	7
Eye		
Right	19	8
Left	10	5
Laterality ^a^		
Unilateral	21	6
Bilateral	5	7
IIRC		
Group D	23	12
Group E	6	1
First signs ^b^		
Leukocoria	16	9
Strabismus	2	0
Erythematous eyelid	2	0
No. of IAC cycles		
Median (minimum-maximum)	3 (2–6)	3 (2–6)
Additional treatments ^c^		
None	13	4
IVC	0	13
Local cryotherapy or laser ablation	14	7
Intravitreal chemotherapy	5	2
Pars plana vitrectomy	4	1

Note: *IVC* = intravenous chemotherapy, *IAC* = intra-arterial chemotherapy; *IIRC*=International Intraocular Retinoblastoma Classification

^a^Both eyes were treated with IAC in three patients

^b^Some patients had more than one sign

^c^Some patients had several additional treatments

**Fig. 1:**
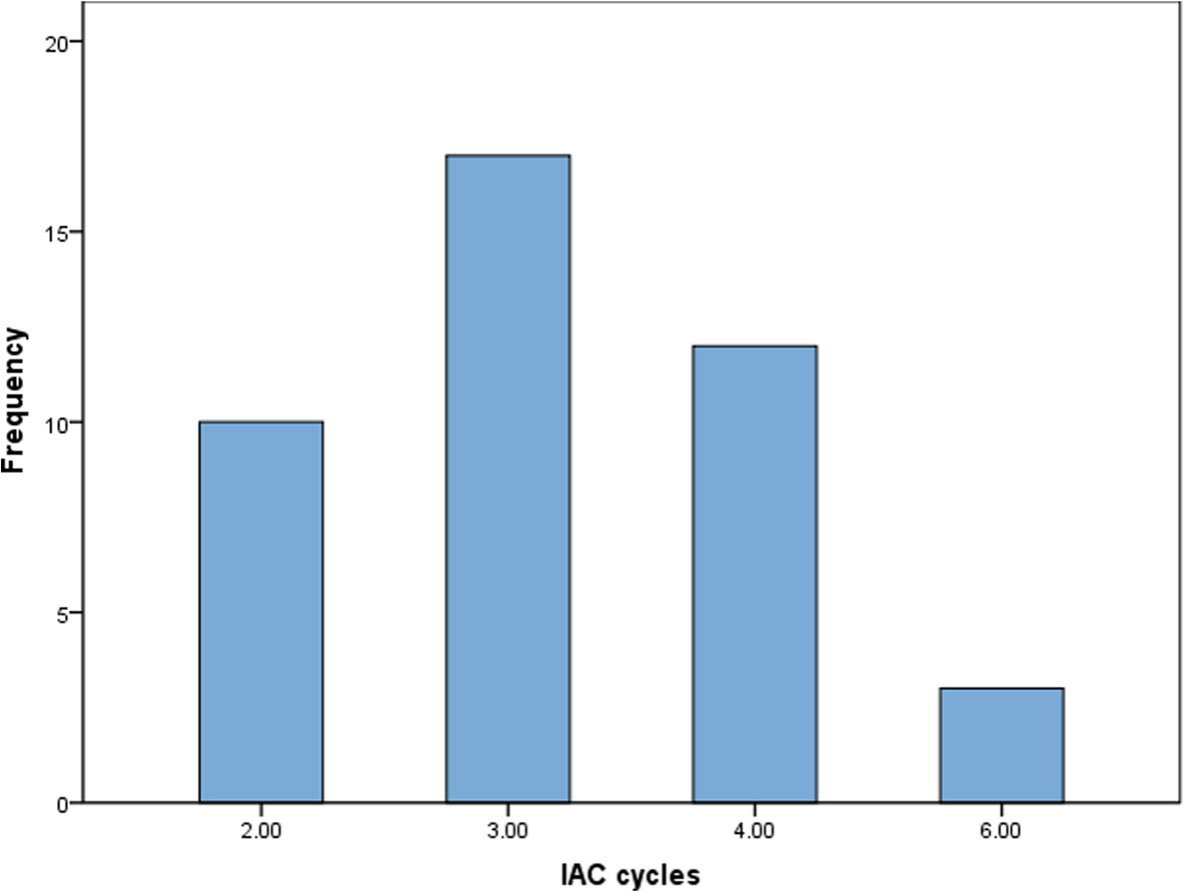
Number of patients receiving different cycles of intra-arterial chemotherapy

A total of 137 IAC procedures were performed, with a mean of 3 sessions per eye (range, 2–6 sessions). Catheterization failure occurred because of vasospasm of the internal carotid artery in a 2-month-old infant. The technical success rate was 99.3% (136/137). The drug dosage ranges were as follows: melphalan, 2.5–5 mg; topotecan, 0.5–1 mg; and carboplatin, 20–40 mg. The chemotherapeutic agent dose for each session was adapted according to the patient’s response to previous IAC cycles. Each IAC cycle was performed after a 3- to 4-week interval (Fig. 1). In the secondary IAC group, all patients received 1 to 6 IVC cycles (median, 3 cycles) at 28-day intervals (Table 2).

**Table 2:** Intra-arterial chemotherapy for advanced retinoblastoma as secondary treatment in 13 eyes

Secondary IAC	No. of Eyes (%)
Indication for IAC	
Tumor recurrence	3 (23.1%)
Disease progression	2 (15.4%)
NA	8 (61.5%)
No. of previous IVC cycles	
1 cycle	2 (15.4%)
2 cycles	4 (30.8%)
3 cycles	5 (38.5%)
6 cycles	2 (15.4%)
Globe salvage	11 (84.6%)

Note: *IVC* = intravenous chemotherapy, *IAC* = intra-arterial chemotherapy

### Survival outcomes

**Fig. 2:**
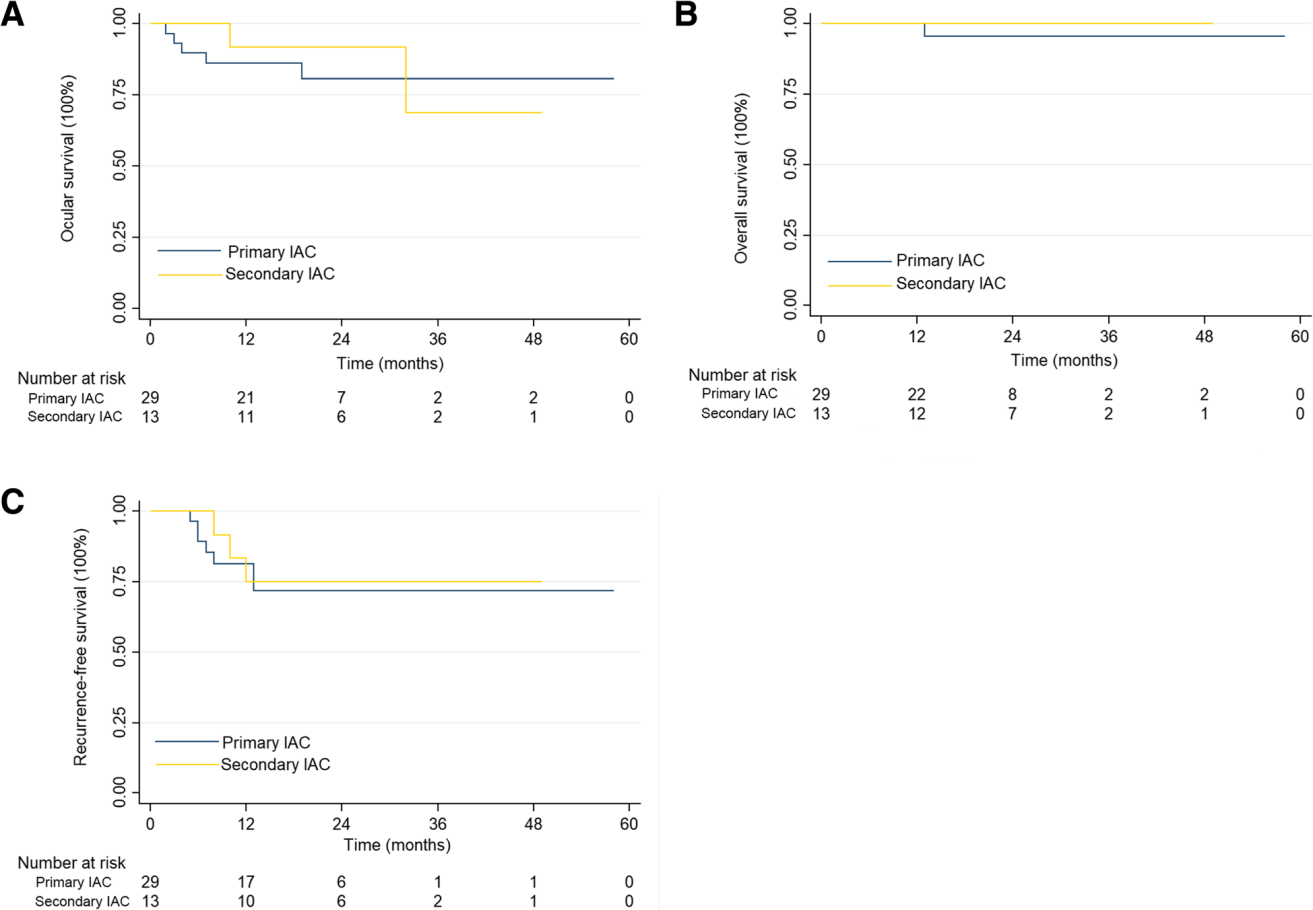
Kaplan-Meier survival curves for IAC as primary or secondary treatment for patients diagnosed with advanced retinoblastoma under three months of age. Ocular survival (**a**), overall survival (**b**), and event-free survival (**c**). IVC = intravenous chemotherapy. IAC = intra-arterial chemotherapy

The survival outcomes for the 2 groups are shown in Table 3. The globe salvage rates for group D and group E eyes treated with primary IAC were 95.7% (22/23) and 33.3% (2/6), respectively, while the overall 2-year ocular survival rate was 80.7% (95% confidence interval [CI], 58.9–91.7; Fig. 2a). Details of eyes treated with secondary IAC are listed in Table 2. In this group, globe salvage was achieved for 83.3% (10/12) of group D eyes and 100% (1/1) of group E eyes. The overall 2-year ocular survival rate was 91.7% (95% CI, 53.9–98.8; Fig. 2a).

**Table 3:** Survival outcomes of 39 infants with advanced retinoblastoma

	Primary IAC	Secondary IAC
Globe salvage	24	11
Ocular survival rate	82.8%	84.6%
Death	1	0
Overall survival rate	96.2%	100%
Recurrence	6	3
Time to recurrence, months ^a^	8.3 ± 3.4	10.0 ± 2.0
Stable disease after salvage treatment ^b^	6	2
Recurrence-free survival rate	79.3%	76.9%

Note: *IVC* = intravenous chemotherapy, *IAC* = intra-arterial chemotherapy

^a^Data were represented as mean ± standard deviation

^b^Salvage treatment included intravenous chemotherapy, intra-arterial chemotherapy, laser ablation, intravitreal chemotherapy, and pars plana vitrectomy

During the follow-up period, 1 patient with unilateral disease (group E eye) in the primary IAC group developed extraocular disease and died. He had received 4 IAC cycles with subsequent enucleation for tumor progression. The interval between enucleation and death was 6 months. No patient developed metastasis, secondary malignancies, or pinealoblastoma. The 2-year overall survival rates were 95.5% (95% CI, 71.9–99.4) and 100% (95% CI, 100.0–100.0) in the primary and secondary IAC groups, respectively (Fig. 2b).

Tumor recurrence occurred in 9 eyes (6 in the primary IAC group and 3 in the secondary IAC group) during the treatment period. The intervals between diagnosis and tumor recurrence were 8.3 ± 3.4 months (mean ± standard deviation) in the primary IAC group and 10.0 ± 2.0 months in the secondary IAC group. The tumor was successfully eliminated after further treatment (laser photocoagulation, plaque radiotherapy, cryotherapy, intravitreal chemotherapy, and pars plana vitrectomy) in all but 1 eye. The 2-year recurrence-free survival rates were 71.9% (95% CI, 49.4–85.7) and 75.0% (95% CI, 40.8–91.2) in the primary and secondary IAC groups, respectively (Fig. 2c).

### Complications

The treatment-related complications are listed in Tables 4 and 5. Some adverse events related to intra-arterial injection were recorded. Ocular adverse events included eyelid erythema in 1 eye and fundus hemorrhage in 5 eyes. Severe ocular adverse events such as cellulitis-like severe orbital inflammation and diffuse chorioretinal atrophy were not observed.

**Table 4:** Adverse events due to intra-arterial injection

Ocular adverse events	No. of eyes (%)
Severe	
Cellulitis-like severe orbital inflammation	None
Diffuse chorioretinal atrophy	None
Mild	
Erythematous eyelid	1 (2.4%)
Fundus hemorrhage	5 (11.9%)
Systemic adverse events	No. of patients (%)
Severe	
Cerebral infarction	None
Myelosuppression	3 (7.7%)
Sepsis	None
Mild	
Intraoperative bradycardia due to the vagal reflex	None
Intraoperative bronchospasm	None
Transient vomiting and hair loss	1 (2.6%)
Transient pancytopenia	1 (2.6%)

Note: ^a^ Some patients had more than one adverse event

**Table 5:** Complications after intra-arterial chemotherapy for advanced retinoblastoma

Complications ^a^	No. of eyes (%)
Eyelid edema	1 (2.4%)
Phthisis bulbi	8 (19.0%)
Leukemia	None
Secondary neoplasms	None
Vision loss	8 (19.0%)
Poor vision	4 (9.5%)
Cataract	1 (2.4%)

Note: ^a^ Some patients had more than one complication

Three patients exhibited different degrees of myelosuppression, although only 1 of them had severe myelosuppression characterized by a significant decline in neutrophilic granulocytes. The condition resolved after subcutaneous injection of granulocyte-stimulating factor. Other systemic adverse events included transient vomiting and hair loss in 1 patient and transient pancytopenia in 1, both of whom showed spontaneous resolution. There were no procedure-related severe systemic complications, such as neurological impairment, stroke, cerebral infarction, sepsis, or limb ischemia.

Given the very young age of patients in this study, it was difficult to determine the Snellen visual acuity. However, vision loss occurred in 8 eyes, while 4 eyes exhibited poor vision at the last recorded follow-up visit. Moreover, phthisis bulbi and cataract were observed in 8 eyes and 1 eye, respectively.

## Discussion

In this retrospective single-center study, we found that IAC, whether primary or secondary, is an effective treatment for advanced intraocular retinoblastoma in infants < 3 months of age, with a technical success rate of 99.3% and globe salvage rate of > 80%. Most eyes were salvaged without the need for enucleation or EBRT, and none of the patients developed metastasis, secondary malignancies, or pinealoblastoma. Thus, the globe salvage and overall survival rates after IAC in our center were better than the outcomes in previous reports of infants treated primarily with radiation therapy or IVC [17]. Although complications, such as phthisis bulbi, vision loss, poor vision, and cataract, occurred in some patients, serious adverse events related to intra-arterial injection were not observed, which suggests that IAC is fairly safe for such small infants.

In our study, 1 patient with unilateral disease (group E eye) in the primary IAC group developed extraocular disease and died. He had received 4 IAC cycles with subsequent enucleation for tumor progression. Histopathological analysis of the enucleated eye revealed high-risk feature of optic nerve invasion. However, he did not receive postenucleation adjuvant chemotherapy to lower the risk of metastasis. There is evidence that IVC as adjuvant postoperative treatment can effectively prevent metastasis in high-risk patients who have undergone enucleation [18]. Therefore, we think it is important to administer postenucleation adjuvant chemotherapy to lower the risk of metastasis in patients with high-risk histopathological features. IVC can provide a prophylactic benefit against metastases, whereas IAC for the direct delivery of drugs to the eye with minimal systemic effects may not offer protection against systemic disease. There is a concern for metastasis when treating germline patients with IAC alone in the absence of IVC. Although none of the patients developed metastasis, secondary malignancies, or pinealoblastoma in our study, considering the short-term follow-up, longer follow-up periods are necessary to assess secondary cancers and mortality.

IAC is an invasive technique that requires repeated placement of an arterial sheath in the femoral artery and catheterizations of the ophthalmic artery. This technique is challenging and requires a clinician who is experienced in interventional neuroradiology or endovascular neurosurgery and comfortable with cannulation into the brain of a toddler or an infant [13]. In neonates and very young infants, whose arteries are just slightly larger than the catheters used, catheterization may not be successful or can result in fatal complications, such as arterial thrombosis or dissection. Our direct microcatheterization technique, which involves advancement of the microcatheter from the femoral sheath to the ophthalmic artery without the help of a larger intermediary catheter, facilitates a decrease in the size of the femoral access sheath. Vasospasm may also be an issue, although clinicians generally wait for it to resolve and proceed with catheterization. On the other hand, emboli from atherosclerotic plaques, which are the biggest causes of neurological complications in adults, is not an issue in young children. In the present study, the technical success rate for IAC was 99.3%, which is consistent with that in previous reports [10, 19, 20].

Although serious complications from the IAC procedure, such as cerebral hemorrhage, brain infarction, arterial thrombosis, and arterial dissection, were not observed in our study, physicians should be aware of the possibility of these serious complications, and this should be understood by the parents of infants undergoing this procedure [21–23]. In addition, precautions are important in protecting each patient, as anatomic variations in the vasculature can also pose a challenge. Therefore, it is important to stress again that the administration of IAC for neonates and very young infants should be performed in experienced centers.

IAC is widely used in the treatment of advanced intraocular retinoblastoma, although there are no unified standard regimens for the chemotherapy drugs. This is particularly true for infant cases, where factors associated with organ development and ontogeny play a major role in the determination of drug disposition. Melphalan, which is not used for systemic chemotherapy because of high toxicity, is the predominant drug used in IAC for retinoblastoma. The dose depends on the age of the patient, and it has been tolerated well when administered intra-arterially at doses < 0.5 mg/kg [24]. In the present study, the drug dosage was calculated based on the patient’s age, which provided an estimate of the eye size and angioanatomy, rather than the body weight [25]. The body weight was used for only limiting the total systemic dose, particularly for melphalan, in the youngest children with bilateral disease [26]. To avoid cumulative systemic toxicity with single-drug chemotherapy, we used a double-drug regimen involving smaller doses of melphalan combined with carboplatin or topotecan. Specifically, the dose of melphalan used in each cycle was < 5 mg for most infants (except in 2 infants), and it was supplemented with topotecan 0.5–1 mg or carboplatin 20–30 mg. Furthermore, to achieve the best drug effects without serious complications, the chemotherapeutic agent dosage was adapted according to the response to the previous IAC cycle. In the present study, 7.7% of cases exhibited grade 1–3 myelosuppression after IAC, while 1 patient each exhibited transient vomiting and hair loss and transient pancytopenia. These complications were associated with the toxicity of the chemotherapy drugs. In addition, 11.9% of eyes exhibited fundus hemorrhage that eventually showed almost complete resolution. This could be related to the concentration of the drugs or the infusion pressure. Another reason could be that drastic calcification of the tumor causes traction on the tumor feeder vessels [12]. Even though the melphalan dosage for infants aged < 3 months is lower than that for older patients, the survival outcomes in our study were similar to those associated with conventional dosages used for older age groups. This study demonstrated that lower doses can be effective in appropriately selected cases.

The preservation of central vision after the early diagnosis of retinoblastoma is particularly challenging because the tumors are often located in the posterior pole, close to the optic disc and macula. Extensive local treatments, such as cryotherapy, laser therapy, and plaque radiotherapy, in these areas can permanently compromise vision [3, 27]. Moreover, complications associated with catheterization and high doses of melphalan may contribute to vision loss [28]. In the present study, cataract, vision loss, and poor vision developed in 1, 8, and 4 eyes, respectively. There was no case of metastasis, and most eyes retained good vision. Our patients were too young to undergo the measurement of visual acuity, which reflects chorioretinal and optic nerve functions, so we assessed visual function based on the following: 1) if the infant’s eyes can follow the moving target; and 2) if the infant appears to frown or close his/her eyes when exposed to strong light. The overall visual outcomes indicated that IAC allows the preservation of retinal function in the short term. A longer follow-up period is necessary to assess the long-term visual outcomes.

This study has some limitations. First, it was a retrospective study with a relatively small sample size. However, retinoblastoma is a rare pediatric tumor, and patients with advanced retinoblastoma diagnosed before 3 months of age are even rarer. Second, the follow-up period of 2 years was relatively short. Although we anticipate that a longer follow-up period will provide more information about the long-term tumor response and visual outcomes, most recurrences after IAC are observed within a year of the last treatment. Finally, vision preservation is an important prognostic factor for evaluation of the efficacy of IAC. Given the very young age of our patients, we could not record the Snellen visual acuity, which is an indicator of chorioretinal and optic nerve functions.

## Conclusions

In conclusion, the findings of this study suggest that IAC, whether primary or secondary, is effective and fairly safe for the management of advanced retinoblastoma in infants aged < 3 months, with a high technical success rate. Despite these favorable initial results, adverse events related to intra-arterial injection and the visual outcomes cannot be neglected and require further investigation. Until the precise benefits and risks of IAC are identified, this treatment should be used with caution in very young infants.

## Data Availability

The datasets used and/or analyzed during the current study are available from the corresponding author on reasonable request.

## Abbreviations

EBRT: External beam radiotherapy
FMA: The femoral artery
IAC: Intra-arterial chemotherapy
IVC: Intravenous chemotherapy
